# Carbon Quantum Dots’ Synthesis with a Strong Chemical Claw for Five Transition Metal Sensing in the Irving–Williams Series

**DOI:** 10.3390/nano12050806

**Published:** 2022-02-27

**Authors:** Anastasia Yakusheva, Anastasia Sayapina, Lev Luchnikov, Dmitry Arkhipov, Gopalu Karunakaran, Denis Kuznetsov

**Affiliations:** 1 Department of Functional Nanosystems and High-Temperature Materials, National University of Science and Technology MISIS, Leninsky Prospect 4, 119049 Moscow, Russia; saiapina.99@mail.ru (A.S.); lyuchnikov.lo@misis.ru (L.L.); hipa2010@yandex.ru (D.A.); dk@misis.ru (D.K.); 2 Institute for Applied Chemistry, Department of Fine Chemistry, Seoul National University of Science and Technology (Seoul Tech), Gongneung-ro 232, Nowon-gu, Seoul 01811, Korea; karunakarang5@gmail.com

**Keywords:** carbon quantum dots, chelating agent, fluorescence properties, fluorescence quenching, fluorescence polarization, surface design, transitional metal’s salts, aminopolycarboxylic acid (APCA)

## Abstract

Carbon quantum dots (CQDs) are an excellent eco-friendly fluorescence material, ideal for various ecological testing systems. Herein, we establish uniform microwave synthesis of the group of carbon quantum dots with specific functionalization of ethylenediamine, diethylenetriamine, and three types of Trilon (A, B and C) with chelate claws -C-NH_3_. CQDs’ properties were studied and applied in order to sense metal cations in an aquatic environment. The results provide the determination of the fluorescence quench in dots by pollutant salts, which dissociate into double-charged ions. In particular, the chemical interactions with CQDs’ surface in the Irving–Williams series (IWs) via functionalization of the negatively charged surface were ascribed. CQD-En and CQD-Dien demonstrated linear fluorescence quenching in high metal cation concentrations. Further, the formation of claws from Trilon A, Trilon B, and C effectively caught the copper and nickel cations from the solution due to the complexation on CQDs’ surface. Moreover, CQD-Trilon C presented chelating properties of the surface and detected five cations (Cu^2+^, Ni^2+^, Ca^2+^, Mg^2+^, Zn^2+^) from 0.5 mg/mL to 1 × 10^−7^ mg/mL in the Irving–William’s series. Dependence was mathematically attributed as an equation (ML regression model) based on the constant of complex formation. The reliability of the data was 0.993 for the training database.

## 1. Introduction

In recent times, applying natural assembled nanomaterials is the primary strategy in eco–build environments [1]. The promising material market strategy is to replace toxic or non-eco-compatible materials and technologies with eco–friendly ones [2]. Thus, carbon quantum dots (CQDs) are zero-dimensional carbon nanomaterials with an amorphous core and functional groups on the surface [3]. They have gained much attention among various emissive dots with quantum effects, such as MeO nanoparticles [4] or semiconduction cadmium, germanium, indium, or zinc-containing zero-dimensional materials [5]. For CQDs, the trend in synthesis was from natural carbon sources, which expanded the market for the dots due to their economic, reproducible, and environmentally friendly peculiarities [6]. This technology opens up new avenues for controlling the natural environment and has been successfully applied in the water assay [7].

Unfortunately, CQDs are sensitive to inhomogeneous nucleation and non-controllable core growth [8]. Foreign impurities and chemical compounds are critical for the CQD core’s structural and physical properties [9,10,11]. According to the literature, precursors with carboxyl, carbonyl, hydroxyl, amino, nitro, or other organic molecular fragments provide valuable information. Surface chemistry correlates with an increase in CQDs’ applicability via tunable photoluminescence, good multi-photoexcitation, stability, and water solubility. Furthermore, the homogeneous functionalization of carbon quantum dots enhances the accuracy and simplicity of measurements [12,13].

One problem here is that the multi-cation compound of the water probe is not an easily detectable cause of the competitive adsorption mechanism on the CQDs’ surface [14,15]. However, the quencher may collaborate with the CQDs’ surface in two ways: via the formation of the nonfluorescent ground-state complex (static quenching) or the redistribution of charge (shift in electron state) between CQDs and the quencher [16].

Careful analysis of the route and conditions among nitrogen-containing CQDs in Table S1 (Supplementary Materials) presents nitrogen inclusion as one of the most effective ways to enhance the dots’ quantum yield and sensibility. Generally, data suggest that using nitrogen sources leads to high (more than 50%) quantum yield, fluorescence stability, and good solubility in water [17]. CQDs with -NH_2_ functionalization represent well-being in sensing applications [18]. Since synthesis, the energy-consuming, long-time hydrothermal method has been predominated by microwave heating, which requires low-energy, low-time demand, and homogeneous irradiation [19]. In the case of sensing, the wide variety of contaminants, such as proteins, vitamins, metal cations, anions, and gases, makes the sensing mechanism unclear [20]. Data show that measurements with different compounds and the surface chemistry of CQDs include disorder in the understanding of properties [21,22,23,24].

Our work was based on the analytical chemistry law in co-ordination compounds and the analysis of N-doped CQDs. For example, the interaction between nitrogen centres in dots and, in the widespread literature, copper or nickel contaminates conditions due to complex formation on the surface [25,26]. Presumably, the physicochemical properties of the surface must correlate with the properties of the precursors. Thus, the denticity of complexion, stability constants, crystal field stabilization energy (CFSE), and cation radius are critical [27]. In theory, the nitrogen-containing ligands with saturated donor bonds show a promising correlation according to the Irwing–Williams series [28], due to complex formation on the surface with various ranges of stability [29].

In this work, ethylenediamine and diethylenetriamine, Trilon A (nitrilotriacetic acid), Trilon B (ethylenediaminetetraacetic acid), and Trilon C (diethylenetriaminepentaacetic acid) as precursors with an amino group, particularly with -C-NH_2_ fragments, were explored. The investigation focused on the co-ordination processes around the CQDs in cationic metal solutions as surface reactivity analysis by fluorescence quenching. The results identify fundamental terms of the CQDs’ surface for sensing applications. We revealed patterns for the carbon core with nitro or Trilon (A, B and C) groups by fast and low-cost microwave heating. The results demonstrate the opportunity to form functional claws from precursors on the CQDs’ surface, and rendered the main properties close to the initial complexing agent.

## 2. Materials and Methods

### 2.1. Reagents and Materials

The first synthesis stage included citric acid (99.8%) and ammonium carbonate (99.0%) as precursors for carbon core formation. The amino and claw functionalization were provided by ethylenediamine (En), diethylenetriamine (Dien), nitrilotriacetic acid (NTA, Trilon A), ethylenediaminetetraacetic acid (EDTA, Trilon B), and diethylenetriaminepentaacetic acid (DTPA, Trilon C) powders. High-purity (99.9%) metal salts such as CuCl_2_, CaCl_2_, MgCl_2_, FeCl_2_, NiCl_2_, ZnCl_2_ were used as pollution contaminants. In all the experiments, deionized water was used as a dispersant.

### 2.2. Synthesis Part

The CQDs with chelate claws and nitro functionalization were synthesized in a two-stage microwave irradiation route. The carbon quantum core was first prepared by mixing highly dispersed powder of citric acid and ammonium carbonate in a ceramic crucible at a 2:1 molar ratio of 6 g (50 mL volume). It was heated for 3 min and 30 s under 700 W of microwave irradiation, and the temperature afterward was near 150–170 °C via an IR thermometer. The dark brown primary product was cooled down to room temperature and then dispersed in 25 mL of deionized water for 10 min in a US-bath treatment (frequency of 35 kHz). The core purification (CQD-CO_3_) was performed by centrifuging at 6000 rpm for 30 min. The final precipitate was dried in the incubator at 60 °C, and the obtained dry residues were milled. Its carbon powder was named CQD-CO_3_.

Then, the functionalization of the CQD-CO_3_ surface was conducted by a similar procedure to the one mentioned above. The powder of CQD-CO_3_ and the functional precursors (En, Dien, and Trilon A, B and C) were mixed at a molar ratio of 3:1. For En precursors, the irradiation time in the second stage was 1 min 20 s. For Dien, the irradiation time was 2 min, while irradiation lasted for 2 min 30 s for all Trilons. Centrifugation time rose to 40 min at 14,500 rpm, and products were saved in dry atmospheric conditions. The purification stage was repeated to recover the liquid and solid-state of CQD and filtered through a Millex-LG 0.2 µm IC filter cartridge (Millipore, Burlington, MA, USA) to achieve the appropriate size quality. Synthesized CQDs were named CQD-En, CQD-Dien, CQD-Trilon A, CQD-Trilon B, and CQD-Trilon C via the names of precursors in the functionalization step, respectively.

### 2.3. Characterization Part

Size distribution was analyzed by dynamic light scattering (DLS) measurements and zeta potentials by Zetasizer Nano ZS (ZEN3600), Malvern (UK). The IR Fourier spectrometer Thermo NICOLET 380 (USA) confirmed the functionalization of CQDs via chelate claws or nitrogen fragments. Moreover, surface behaviour in water was completed by zeta potential measurements by Zetasizer Nano ZS (ZEN3600), Malvern (UK) again.

A summary of the optical effects due to the different CQDs’ core functionalizations was compared via the absorption spectrum from a spectrophotometer UV mini-1240 Shimadzu (Japan) in the 250–500 nm range. Massive fluorescence data was characterized by fluorescence intensity and stability in time from Agilent Technologies’ Cary Eclipse Fluorescence Spectrophotometer (USA).

The calculation for CQDs’metal sensing and the creation of the lineal prediction model in the Irving–Williams series were obtained using the OriginLab program and the machine learning method in Python 3.10, respectively.

### 2.4. Detection of Metal Ions by CQDs

In the measurement procedure, the tubes with salt solutions in the goal range (from 1 or 0.5 mg/mL, depending on the solubility, to 1 × 10^−9^ mg/mL via diluting) were prepared. The sensing ability of CQDs was investigated via continuous measurements with the following salts as the cations from the Irving–Williams series: CuCl_2_, NiCl_2_, CaCl_2_, MgCl_2_, FeCl_2_, ZnCl_2_. Analytical measurements of the water probe by CQDs were provided by the FL measurements concerning water contamination. In order to have reproducible data, we took the volume of the CQDs’ dispersion for the Fl_max_ intensity (1000 a.u.). The measurements were performed by taking 1 mL of diluted salts in a cuvette with the respective amount of CQDs (in μL), and then the FL was recorded. At 0.05 mg/mL concentration, all dots (CQD-En, CQD-Dien, CQD-Trilon A, CQD-Trilon B, and CQD-Trilon C) were used and saved.

### 2.5. The Calculation of the Calibration Curve and the Model of Linear Regression

CQDs’ optical properties were correlated with the fluorescence quenching effects of metal cations [30]. The FI was normalized to the maximum (1000 a.u. of FI) for each calibration curve and gave the coefficient behind the concentration data by linear regression tools. A polynomial equation was created. Next, for the assumption of the ability of the CQDs’ surface to establish chelate complex, we created the training dataset as the date of the added different types and concentrations of divalent cations in the probe with the CQDs. The result was assumed to approximate the original (primary) equation, and the validity of the data was assessed.

## 3. Results

### 3.1. Characterization of CQD-CO_3_ and CQD-En, Dien, Trilon A, B, C Functionalized Particles

**Figure 1:**
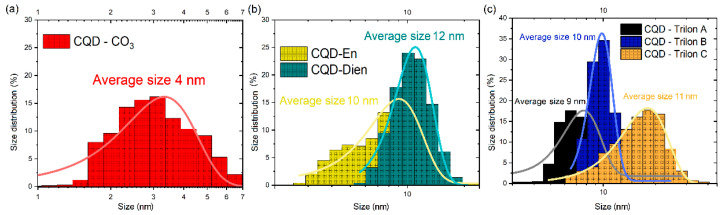
The size distribution of (**a**) CQD-CO_3_, (**b**) CQD-En, Dien, and (**c**) CQD-Trilon A, B, C via dynamic light scattering measurements.

To further explore the significant influence of functionalization and nano-dimension morphology, size distribution was calculated via the dynamic light scattering (DLS) function, which provides a mean size of CQDs in normal distribution fitting in Figure 1.

CQD-CO_3_ in Figure 1a shows a narrow size, with an average of 4 nm in diameter. Furthermore, the distributive trends represent the shift in the average size for CQD-En at 10 nm and 12 nm for CQD-Dien, respectively. CQD-Trilons were superimposed step by step. The peaks were from 7–9 nm for CQD-Trilon A to 16–20 nm for CQD-Trilon C. Noticeably, the size was probably correlated with the molecular weight of the precursors, and CQD-Trilon C has the largest particle size.

### 3.2. The Assumption in Claw Formation on CQDs’ Surface

Understanding the formation and growth of nanodots is critical for achieving comparable results. Decomposition processes were based on the kinetics of the thermoactivated chemical reaction in time [8,19]. Based on the physical properties of the precursors, we concluded that the synthesis temperature of 150–170 °C was too soft for the incomplete carbonization process. Furthermore, the thermal decomposition of precursors corresponded with the activation energy lever in chemistry bond destruction. According to table data, En and Dien lost structure via to the low energy C–N bond (293 kJ/mol). Further molecular reorganization occurs at the polar covalent bonds of 390 and 414 kJ/mol between hydrogen and nitrogen or hydrogen and carbon bands.

**Figure 2:**
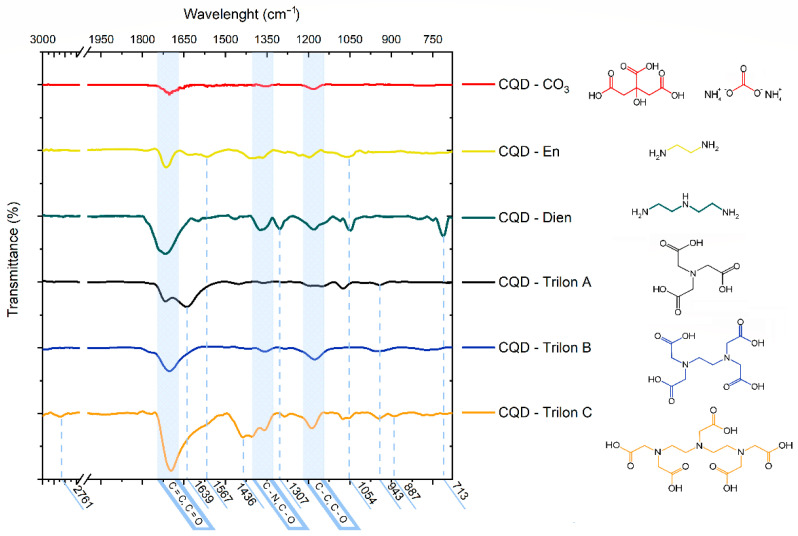
The mid-infrared (MIR) spectroscopy measurements of a carbon core in comparison with the functionalized CQD-En, Dien, and CQD-Trilon A, B, and C samples.

The Trilons involved had oxygen-containing groups such as C=O (708 kJ/mol), C–O (344 kJ/mol), and O–H (460 kJ/mol). The degradation process started with a low-energy single bond between C–N and C–O. Nevertheless, the higher energy carbon–carbon chain and carboxyl groups were saved. The practical explanation for organic bonds in the CQD family related to mid-infrared (MIR) spectroscopy results in Figure 2.

The dry CQD core and CQD-En, Dien, and Trilon A, B, and C were measured. Chemical bonds were compared with the FT-IR spectra of the reference peak position from precursors in Figure S1 (Supplementary Materials) and functional molecules in Figure 2.

The peak in the lower wavelength region characterizes the carbon–carbon, carbon–oxygen, and carbon–nitrogen molecular bonds [31]. These three peaks mentioned above are common to all CQD types. The clustering structure shows the sp^3^-hybridization forms in the C–C and C–O bonds as peaks at 1230–1120 cm^−1^ [32]. The right side of the peak was attributed to carbon and the left side to the carbon–nitrogen pair. The second peak in the CQD-CO_3_ spectra corresponds to the C–O bond at 1384 cm^−1^ and the broadening double peaks from the C–N bond at 1320 cm^−1^. The oxidative carbon bond (C = O) and the sp^2^-hybridization of C = C are related to the broad peak at 1704 cm^−1^, which was only revealed after carbonization in Figure S1. Hydrogen-containing groups in the 3500–3300 cm^−1^ region were not found via dehydrated samples [33,34,35].

Amino-functionalization was presented as a peak at 1567 cm^−1^ for N = O and 1054 cm^−1^ for C–N from the En spectrum. In the CQD-Dien line, the peak assumes the 1307 cm^−1^ C–C bond from Dien and the band at 713 cm^−1^ due to ammonium carbonate N–H (in Figure S1, (NH_4_)_2_CO_3_ and Dien lines, respectively). CQDs with Trilon A additives started with an IR-black line with an original peak at 943 cm^−1^ and 1065 cm^−1^. CQD-Trilon B supposes a similar peak position. Conversely, the toothy line of Trilon C shows the apparent effect of N–H bending vibration at 1639 cm^−1^ and more than one peak at 943 cm^−1^ for N–O bands. The most informative spectra of CQD-Trilon C additionally have a peak at 2761 cm^−1^, corresponding to C–H stretching vibrations, and at 1436 cm^−1^ related to the stretching band of C–N. The mass of amino-functionalization increased in the range from CQD-CO_3_ to CQD-Dien, and CQD-Trilon A, B, and C were depicted with many minor features in different positions and counts of peaks.

### 3.3. ABS Spectroscopy for CQDs—Family

**Figure 3:**
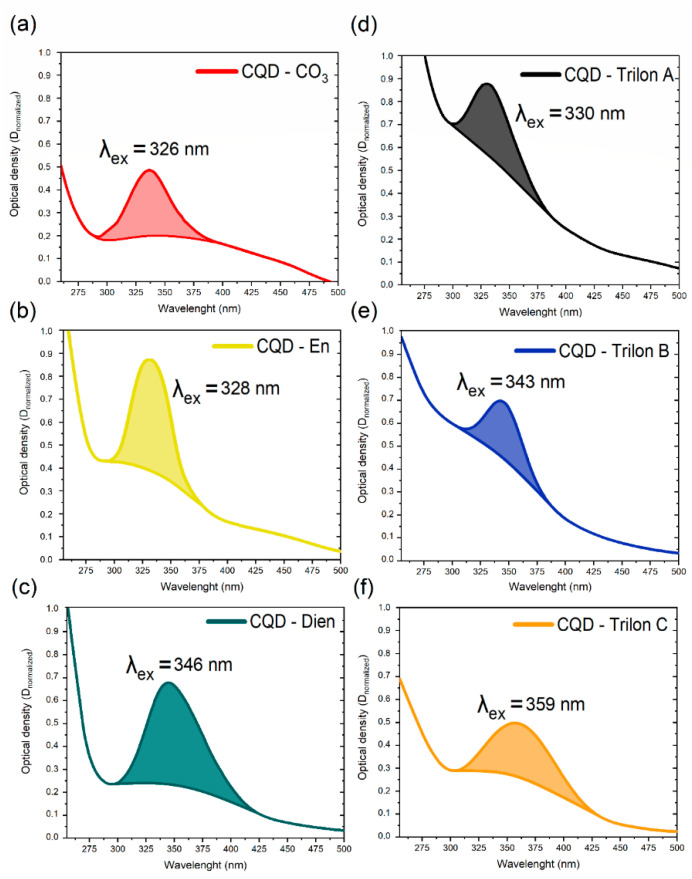
The absorbance spectra of (**a**) CQD-CO_3_, (**b**) CQD-En, (**c**) -Dien, (**d**) Trilon A, (**e**) Trilon B, and (**f**) Trilon C.

The efficacy of absorbance properties was evaluated as proof that the aqueous dispersion of CQDs would be used in sensing. The result was attributed to the absorbance (ABS) spectra in Figure 3.

The bonding theory of visible absorption suggests analyzing the shifted peaks’ positions as a representative factor in the case of the functional group [36]. Figure 3 presents a saturated organic bond like C = C or C = O with π→π ∗ a jump in multi-molecular nuclear. The low energy n→π ∗ jump influences the spectral background in the long-wavelength region for all CQDs. In particular, the characterized peaks show specific functional conjugation with a monosaturated bond. The core of CQDs has a proportional ratio between background and peaks. In contrast, CQD-En has a bright peak at 328 nm, and CQD-Dien has an 18 nm shift in the long-wavelength region due to the changes in π→π ∗ via the joint electron pair in the carbon–nitrogen group. The Trilon group started with the CQD-Trilon A peak at 330 nm, and the effects of the carboxyl group with C = O bonds via vast background squares near 300 nm (the n→−π ∗ the transition of electrons) in particles were noticed. The relative shifts of 13 nm and 29 nm for CQD-Trilon B and CQD-Trilon C were attributed to nitrogen atoms, as with CQD-En and Dien. Hence, the hydroxyl group introduces more electron orbital changes in conjugated systems. In summary, the redshift in the peak position is related to the ratio between functional groups [37] and prevalent surface states [38].

### 3.4. Fl Spectroscopy for CQDs—Family

The most likely cause of the CQDs’ sensing platform is the fluorescence properties. Furthermore, FL intensity and stability are critical for CQDs’ applications [39].

The CQDs were appreciated with relative fluorescence intensity via fluorescence stability in Figure S2 (Supplementary Materials) for CQDs at a 0.05 mg/mL concentration in water. CQD-Trilon B and -Trilon C peaks showed an intensity of three times the size of the other dots. Furthermore, we confirmed that the functionalization-free CQD-CO_3_ core had a lower varying intensity among groups via high-cation mobility in the double electron layer than Zeta-potential measurements in Figure S3 (Supplementary Materials).

**Figure 4:**
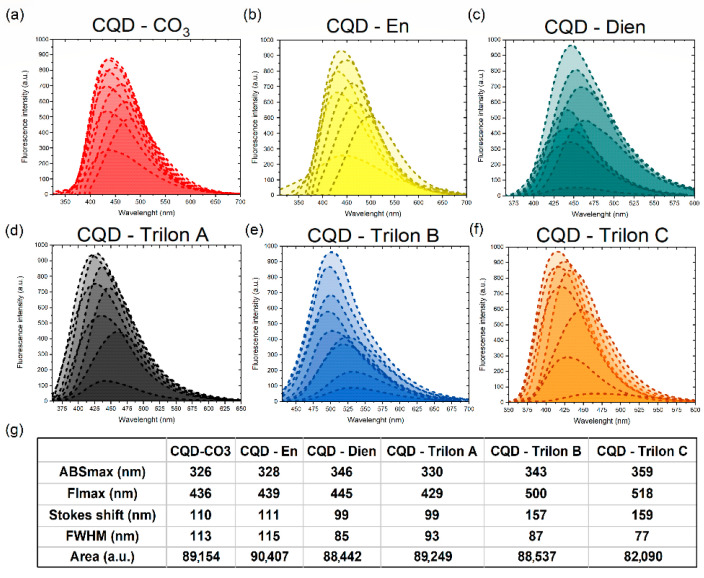
The integrated fluorescence spectra of (**a**) CQD-CO_3_, (**b**)CQD-En, (**c**) -Dien, (**d**) Trilon A, (**e**) Trilon B, and (**f**) Trilon C with the shape parameters of spectra (**g**).

The integration fluorescence spectra were a sum of Fl peaks via measurements of a 10 nm shift in excitation wavelength in Figure 4.

Figure 4 represents the peak position of CQD-CO_3_ compared to functionalized CQDs across all measurements. The Gaussian function recognizes the integrated fluorescence spectra as an order indicator for each CQDs’ surface [40]. Surface functionalization trap states and double electron layer charges are synergetic parameters for various CQDs’ fluorescence responses. Herein, we analyze the FWHM and Stokes shift as surface-sensitive functions. The CQDs’ core and CQD-En had a broad FWHM and significant Stokes shift from the ABS peak position, to which we posit is due to poor functionalization. Next, CQD-Dien had a closer FWHW, and the peak of FL shifted relatively to the previous 445 nm. CQD-Trilon A saves the Stokes shift data, but the FL_max_ has a blueshift to 429 nm. Oppositely, CQD-Trilon B and C include easily hydrolyzed carbocyclic groups in sufficient quantity for a narrow FWHM and a shift of 157 and 159 nm. Thus, we assume an increase in the ratio between nitrogen and carboxylic groups on CQDs makes FL properties more inhomogenous.

## 4. Discussion

### The Relationships of CQDs’ Surface in Ionic Solutions

The theory that CQDs with chelating chemical claws have discrete chemical reactivity properties by metals with different electron radii and ionic mobilities was investigated in this paper. Thus, to study the kinetics of the interaction of doubly charged metal cations, the Irving–Williams series (Ca^2+^ < Mg^2+^ < Fe^2+^ < Ni^2+^ < Cu^2+^ > Zn^2+^) was used. The idea is based on the correlation between the strength of the complex and the ion analysis of transition metals.

**Figure 5:**
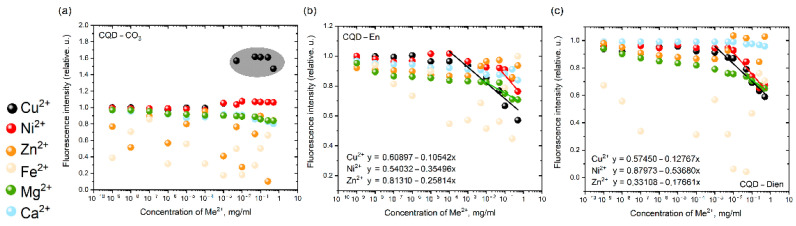
The fluorescence intensity quenching of (**a**) CQD-CO_3_ and (**b**) CQD-En and (**c**) CQD-Dien via reactions with double cation metals from the Irving–Williams series.

All CQD groups show the consequent relations in the IW series from one cation to another. In the cases of CQD-CO_3_ and -En and -Dien in fluorescence intensity measurements, the main trends are determined in Figure 5. It is easy to check that the unfunctionalized carbon core does not have a sensing application. Furthermore, the positively charged surface (Figure S3) of CQD-CO_3_ demonstrates an enhanced intensity of 58% in the middle when we introduced copper ions into the solution. A slightly similar effect was attributed to nickel cations. To the best of our knowledge, fluorescence occurs because of the chlorine anion on the core surface as a concentrator of electrons.

CQD-En and -Dien demonstrate the sensibility due to chemical absorption in the high concentration rate of copper, nickel, and zinc cations in the Irving–Williams series.

**Figure 6:**
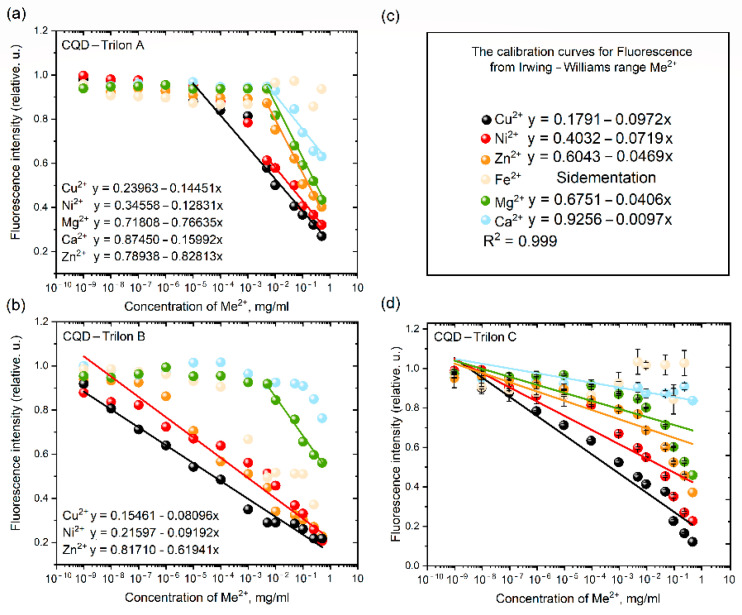
The fluorescence intensity of (**a**) CQD-Trilon A, (**b**) CQD-Trilon B, (**d**) CQD-Trilon C via reactions with double cation metals from the Irving–Williams series and (**c**) the lineal equation for CQD–Trilon C curves via Me^2+^ quenching.

As a result, Figure 6a,b,d depict the values of FI as a function of CQD-Trilon A, B, and C reactivity, which exhibit the most notable changes in reactivity to different metals. The experimental results strongly support the equilibrium properties of the chemical adsorption of copper and nickel cations on the CQD-Trilon A, B, and C surfaces, especially for CQD-Trilon C.

Starting with mathematics, linear regression (machine learning approach) describes the optical properties of nanoparticles due to an increase in the tilt angle as a function of metal cation binding in the Irving–Williams series. In terms of our aim, we created and executed the model to determine the complex composition of the water probe. From this measurement, the initial coefficients of the lineal concentration line were established as follows in Equation (1):

Y(FL) = 0.1186X_Cu_ + 0.0893X_Ni_ + 0.0675X_Zn_ + 0.0567X_Fe_ + 0.0302X_Mg_ + 0.0134X_Ca_ (1)

The introduced-found method carried out the refinement of the model coefficients for several concentrations by adding a known amount of each of the five salts to deionized water. In the case of 20 probes, a different ratio of Me^2+^ cations was explored. The matrix of values made it possible to establish the coefficients of Equation (2) at the next level:

Y(FL) = 10X_Cu_ + 7.5X_Ni_ + 5.5X_Zn_ + 4.3X_Fe_ + 2.5X_Mg_ + 1.1X_Ca_ (2)

The reliability of the data in the training dataset for the IWs was 0.993 for practical applications.

## 5. Conclusions

The general case of our investigation focused on the surface behaviour of CQDs in water systems and the assumption that modified CQDs show the ability to sense contaminants due to analytical chemistry rules. Mainly, sewing chelating claws on the surface demonstrates a strong correlation with complexation laws, particularly the reactivity dependence on the Irving–Williams series.

The results established that the nitrogen-containing CQD surfaces (CQD-En and -Dien) were brightly revealed with the copper in solution. In IWs, CQD-Trilon A and B display the best correlation with the most stable complexation cations, Cu^2+^ and Ni^2+^. Moreover, the most brilliant results were attributed to CQD-Trilon C. CQD-Trilon C showed the best separation in FI properties in the metal ranges as a function of the reactivity of the surface, and was equivalent to the mass of the coefficient before the concentration in the model equation. Thus, the CQD-Trilon C surface in solutions with the same number of cations will form ten chains with copper and only one chain with the calcium cation. Furthermore, the separate detection demonstrates the sensing ability from 0.5 mg/mL to 1 × 10^−7^ mg/mL range for CQD-Trilon C, which presents the best of the results due to chemical claws for five transition metal sensing in the Irving–Williams series.

The presented results from CQD-Trilon C create the basis for the appropriate way to synthesize CQDs with specific chelate surfaces from another chelating agent. The future strategy for CQDs will investigate the physical-chemical parameters of the surface in detail for divalent metal sensing in complex, natural water systems.

## Data Availability

Not Applicable.

## Supplementary Materials

The following supporting information can be downloaded at: https://www.mdpi.com/article/10.3390/nano12050806/s1: Figure S1: The mid-infrared (MIR) spectroscopy table data of peak position for the pure citric acid, (NH_4_)_2_CO_3_, ethylenediamine (En), diethylenetriamine (Dien), nitrilotriacetic acid (NTA, Trilon A), ethylenediaminetetraacetic acid (EDTA, Trilon B), and diethylenetriaminepentaacetic acid (Trilon C); Figure S2: The stability measurements of fluorescence intensity of CQDs in 0.05 mg/mL concentration; Figure S3: The Zeta-potential data of CQD dispersion; Table S1: CQDs literature overview via characteristic properties and application of dots from ethylenediamine, diethylenetriamine, and three types of Trilon (A, B and C) precursors [33,36,41,42,43,44,45,46,47,48,49,50,51,52,53,54,55,56,57,58,59,60,61,62,63,64,65,66,67,68,69,70,71,72,73,74,75].
