# Supplementary material for: Carbon Quantum Dots’ Synthesis with a Strong Chemical Claw for Five Transition Metal Sensing in the Irving–Williams Series
Source: Nanomaterials (Basel). 2022 Feb 27;12(5):806. doi: 10.3390/nano12050806 (PMC8912369; doi:10.3390/nano12050806)
Supplement: Supplementary file 1 [file nanomaterials-12-00806-s001.zip › nanomaterials-1557713-supplementary.pdf]

nanomaterials

MDPI

Supplementary Materials

Carbon Quantum Dots' Synthesis with a Strong Chemical Claw for Five Transition Metal Sensing in the Irving–Williams Series

Anastasia Yakusheva [1,*], Anastasia Sayapina [1], Lev Luchnikov [1], Dmitry Arkhipov [1], Gopalu Karunakaran [2] and Denis Kuznetsov [1]

[1] Department of Functional Nanosystems and High-Temperature Materials, National University of Science and Technology MISIS, Leninsky Prospect 4, 119049 Moscow, Russia; saiapina.99@mail.ru (A.S.) lyuchnikov.lo@misis.ru (L.L.); hipa2010@yandex.ru (D.A.); dk@misis.ru (D.K.)

[2] Institute for Applied Chemistry, Department of Fine Chemistry, Seoul National University of Science and Technology (Seoul Tech), Gongneung-ro 232, Nowon-gu, Seoul 01811, Korea; karunakarang5@gmail.com

* Correspondence: yakusheva.as@misis.ru; Tel.: +7-909-669-3081

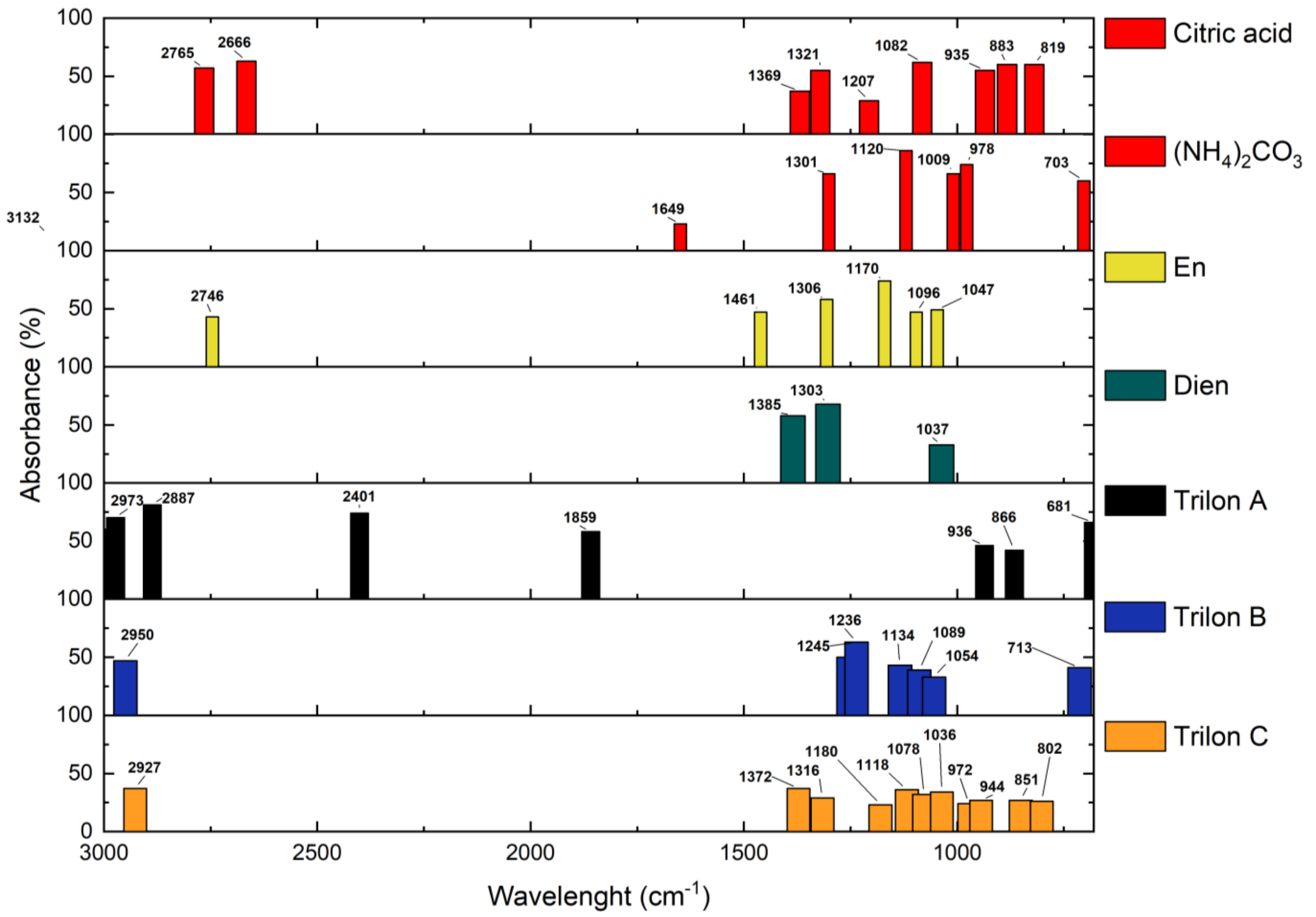

Figure S1. The Mid-infrared (MIR) spectroscopy table data of peak position for the pure citric acid, $(NH_4)_2CO_3$, ethylenediamine (En), diethylenetriamine (Dien), nitrilotriacetic acid (NTA, Trilon A), ethylenediaminetetraacetic acid (EDTA, Trilon B), and diethylenetriaminepentaacetic acid (Trilon C).

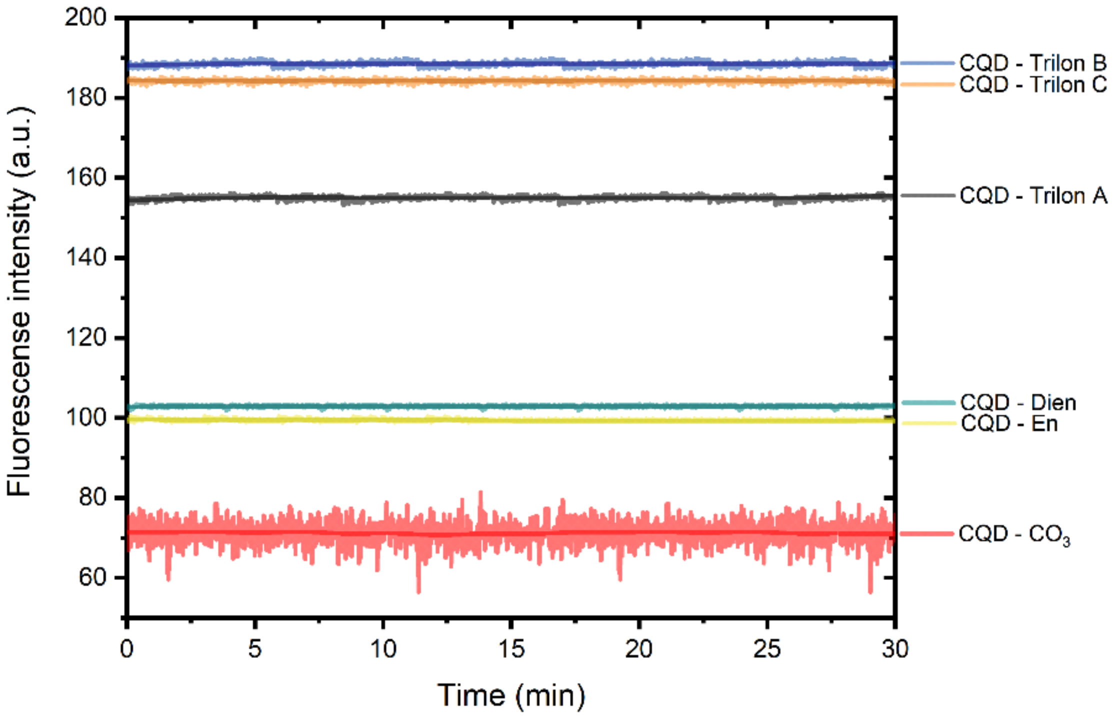

Figure S2. The stability measurements of fluorescence intensity of CQD in 0.05 mg/ml concentration.

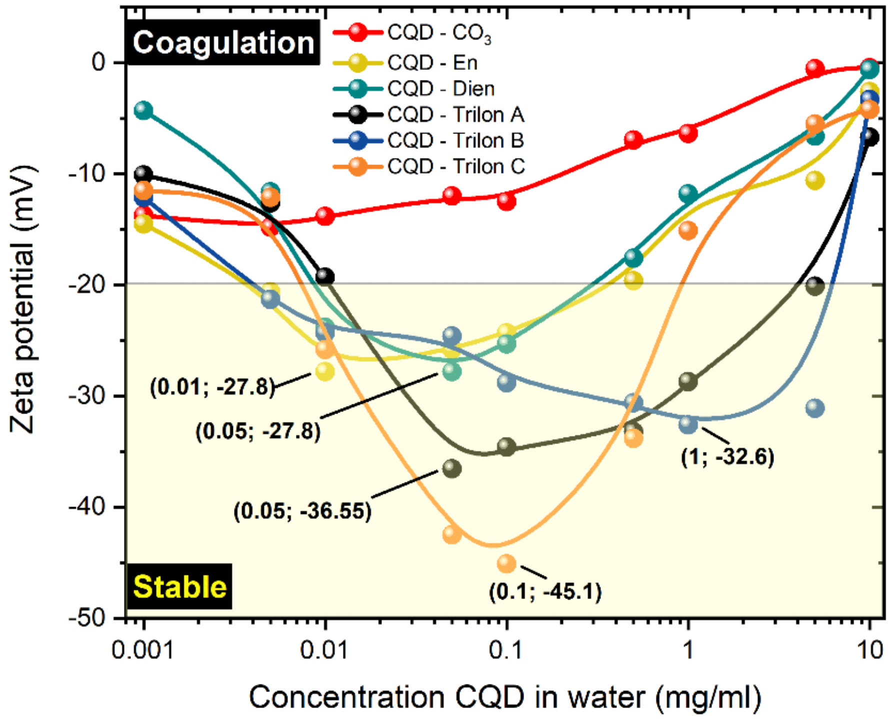

Figure S3. The Zeta-potential data of CQD dispersion.

nanomaterials MDPI

Table S1. The CQDs literature overview via characteristic properties and application of dots from ethylenediamine, diethylenetriamine, and three types of Trilon (A, B and C) precursors.

Article	Precursirs	Synthesis route	Size, nm	PL, nm	QYs	Application	Aplication in particular	Functional groups		
								OH	NH_2	COOH
[1]	Ethylenediamine, alanine	hydrothermal method	8	390	46.2%	biolabeling and bioimaging	**Sensing dihydronicotin-amide adenine dinucleo-tide (NADH)**	1	1	1
[2]	Ethylenediamine, bike soot	hydrothermal method	5.6 nm	396	12.7%	sensing	**Trinitrotoluene**	1	1	1
[3]	Ethylenediamine,citric acid, boronic acid	hydrothermal method	4.5 nm	447	no inf	medisine	**Inhibition of HCoV-229E (COVID)**	1	1	1
[4]	Ethylenediamine, 2,7-dihydroxynaphthalene	hydrothermal method	3.31 nm	493	70.90%	light sourse	**Light emitting diodes**	1	1	1
[5]	Ethylenediamine, ammonium citrate	hydrothermal method	4.8 nm	445	66.8%	sensing	**Sensing Hg2+**	1	1	1
[6]	Ethylenediamine, mandelic acid	hydrothermal method	2.5 nm	429	41.4%.	sensing	**Detection of picric acid**	1	1	1
[7]	Ethylenediamine, microcrystalline cellulose	hydrothermal method	3.2 nm.	360	51%	sensing	**Detection of Fe3+ ions in an acidic environment**	1	1	1
[8]	Ethylenediamine, thiosalicylic acid	hydrothermal method	2.3 nm	420	51.4%	light sourse	**White light emitting diodes**	1	1	1
[9]	Ethylenediamine, citric acid	hydrothermal method	6.7 nm	450	no inf	sensing	**Fe3+ ion detection**	1	1	1
[10]	Ethylenediamine citric acid, polyethylenimine	termoactivated carbonization	1.45 nm	445	no inf	sensing	**Protein sensing**	1	1	1

[11]	Diethylenetriamine, Graphen QD	pulsed laser ablation	3.4 nm	450	66%	investigation of properties	**no inf**	1	1	1
[12]	Diethylenetriamine, citric acid	hydrothermal method	4.5 nm.	431	95%	catalyst	**Photocatalytic performance**	1	1	1
[13]	Diethylenetriamine, citric acid	hydrothermal method	3.16 nm	440	84.79%	sensing	**Selectively detecting ellagic acid**	1	1	1
[14]	Diethylenetriamine, aspartic acid, phosphoric acid,	hydrothermal method	4.2 nm	410	73%.	sensing	**Detection of Cr (VI) and ascorbic acid**	1	1	1
[15]	Diethylenetriamine, Trisodium citrate dihydrate	hydrothermal method	3.5 nm.	450	43%	sensing	**Quantification of Vitamin B12**	1	1	1
[16]	Diethylenetriamine, citric acid	hydrothermal method	10 nm	462	no inf	sensing	**Fluorescent detection of acidic gas**	1	1	1
[17]	Diethylenetriamine, citric acid, urotropine	hydrothermal method	11 nm	451	no inf	sensing	**Sensitive Hg2+ detection**	1	1	1
[18]	Diethylenetriamine, citric acid	microwave method	4–10 nm	450	no inf	chemistry	**Corrosion Inhibition**	1	1	1
[19]	Diethylenetriamine, citric acid	thermal synthesis	4.6 nm	456	11.7%	sensing	**pH-sensitive carbon quantum dots**	1	1	1
[20]	Diethylenetriamine, D(+)-glucose, ethanol, chloral hydrate	chemical route	2–5 nm	455	no inf	biological	**Antimicrobial agent**	1	1	1
[21]	Nitrilotriacetic acid	hydrothermal method	3.7 nm.	440	no inf	sensing	**Folic acid (FA) in human serum samples**	1	1	1
[22]	Nitrilotriacetic acid	hydrothermal method	29 nm.	462	45.8%	investigation of properties	**no inf**	1	1	0
[23]	Nitrilotriacetic acid, ortho-phenylenediamine	hydrothermal method	2.6 nm	450	no inf	sensing	**Colorimetric sensors for water and humidity**	1	1	1

[24]	Ethylenediaminetetraacetic acid disodium salt, $CuCl_2$ •$2H_2O$	hydrothermal method	4 nm	480	54%	sensing	**Sensing of hydrogen sulfide**	1	1	1
[25]	Ethylenediaminetetraacetic acid, ascorbic acid, vitamin C,	microwave method	7-10 mn	490	42%,	sensing	**Dopamine Detection**	1	1	1
[26]	Ethylenediaminetetraacetic acid, 4, 7, 10-trioxa-1, 13-tridecanediamine	microwave method	20 nm	475.4	53.3%	sensing	**Metal ion sensor**	1	1	1
[27]	Ethylenediaminetetraacetic acid, ascorbic acid,	microwave method	10 nm	450	42%	sensing	**CuO and Cu2O by fluorescence quenching**	1	1	1
[28]	Ethylenediaminetetraacetic acid, $Fe(NO_3)_3·9H_2O$,	hydrothermal method	3.5 nm	430	16.23%	sensing	**Dopamine sensing**	1	1	1
[29]	Diethylenetriaminepentaacetic acid (DTPA), Glucose anhydrous, or Metformin hydrochloride (MH)	hydrothermal method (self-polimerization)	3.58 nm.	430	34.23%	investigation of properties	**no inf**	1	1	1
[30]	Diethylenetriaminepentaacetic acid (DTPA), acetic anhydride, pyridine		5.0 nm	460	5.2%	imaging	**Fluorescence and magnetic resonance dual-modal bioimaging**	1	1	1
[31]	Diethylenetriaminepentaacetic acid (DTPA)	heating process	2.5 nm	420	no inf	sensing	**Protein detection**	1	1	1
[32]	Diethylenetriaminepentaacetic acid (DTPA)	heating process	2 nm.	429	no inf	sensing	**Electrochemiluminescent Immunosensor**	1	1	1
[33]	Diethylenetriaminepentaacetic acid (DTPA), p-phenylenediamine, GdCl3·6H2O,	microwave method	2-5 nm	440 to 560 nm	5.76%	imaging	**Fluorescence-MR dual-modality applications**	1	1	1

[34]	Diethylenetriaminepentaacetic acid (DTPA), graphite	laser irradiation	15 nm	400	no inf	investigation of properties	**no inf**	1	1	1
[35]	Diethylenetriaminepentaacetic acid (DTPA), NaOH aque-ous solution	hydrothermal method	3–7 nm	454	4.04%	sensing	**Sensing nitrophenols**	1	1	1
[36]	Diethylenetriaminepentaacetic acid (DTPA), Fe $(NO_3)_3$•$9H_2O$	hydrothermal method	4–6 nm.	450	no inf	sensing	**Ratiometric fluorescence and colorimetric dual-mode assay for H2O2 and xanthine**	1	1	1
[37]	Diethylenetriaminepentaacetic acid (DTPA) powder	heating	5 nm	420	17%	sensing	**Determination of curcumin based**	1	1	1

References for Table S1

[1] A. W. Niu, Y. Li, and R. Z. Dan, Ethylenediamine-Assisted Hydrothermal Synthesis of Nitrogen-Doped Carbon Quantum Dots as Fluorescent Probes for Sensitive Biosensing and Bioimaging, "Sensors and Actuators B" 2015, http://dx.doi.org/10.1016/j.snb.2015.05.006

[2] S. Devi, R. K. Gupta, et. al. "Ethylenediamine mediated luminescence enhancement of pollutant derivatized carbon quantum dots for intracellular trinitrotoluene detection : soot to shine" *RSC Adv.*, vol. 8, pp. 32684–32694, 2018 DOI: 10.1039/c8ra06460a

[3] A. Barras *et al.*, "Functional Carbon Quantum Dots as Medical Countermeasures to Human Coronavirus," ACS Appl. Mater. Interfaces 11, 42964–42974, 2019. DOI: 10.1021/acsami.9b15032

[4] J. Zheng, Y. Xie, Y. Wei, Y. Yang, and X. Liu, "An Efficient Synthesis and Photoelectric Properties of Green Carbon Quantum Dots with High Fluorescent Quantum Yield," Nanomaterials pp. 1–15, 2020. doi:10.3390/nano10010082

[5] V. A. Online *et al.*, "Highly luminescent nitrogen-doped carbon quantum dots as e ff ective fl uorescent probes for mercuric and iodide ions" J. Mater. Chem. C, 2015. DOI: 10.1039/c4tc02756f

[6] Yingte Wang, Xiaoyue Chang, Na Jing and Yong ZhangA. "Hydrothermal synthesis of carbon quantum dots as fluorescent probes for sensitive and rapid detection of picric acid" "Analytical Methods," 2018. DOI: 10.1039/C8AY00441B

[7] P. Wu, W. Li, Q. Wu, and S. Liu, "Hydrothermal synthesis of nitrogen-doped carbon quantum dots from microcrystalline cellulose for the detection of Fe 3 + ions in an acidic environment," *RSC Adv.*, vol. 7, pp. 44144–44153, 2017. DOI: 10.1039/c7ra08400e

[8] X. T. Feng, F. Zhang, Y. L. Wang, Y. Zhang, Y. Z. Yang, and X. G. Liu, "Luminescent carbon quantum dots with high quantum yield as a single white converter for white light emitting diodes," vol. 261106261106, no. 2014, pp. 1–6, 2015. http://dx.doi.org/10.1063/1.4936234

[9] A. I. Journal, S. Devi, A. Kaur, S. Sarkar, S. Vohra, and S. Tyagi, "Synthesis and characterization of highly luminescent N-doped carbon quantum dots for metal ion sensing," *Integr. Ferroelectr.*, vol. 186, no. 1, pp. 32–39, 2018. https://doi.org/10.1080/10584587.2017.1369322

[10] R. M. Freire, N. D. B. Le, Z. Jiang, C. S. Kim, V. M. Rotello, and P. B. A. Fechine, "NH 2 -rich Carbon Quantum Dots : A protein-responsive probe for detection and identification," *Sensors Actuators B. Chem.*, 2017. http://dx.doi.org/10.1016/j.snb.2017.09.085

[11] AS. R. M. Santiago, T. N. Lin, C. H. Chang, Y. A. Wong, C. A. J. Lin, C. T. Yuan, J. L. Shen." Synthesis of N-doped Graphene Quantum Dots by Pulsed Laser Ablation with Diethylenetriamine (DETA) and Their Photoluminescence" ,Physical Chemistry Chemical Physics, DOI: 10.1039/C7CP03993J.

[12] A. Aghamali, M. Khosravi, N. Modirshahla, and A. Mohammad, "Synthesis and characterization of high efficient

photoluminescent sunlight driven photocatalyst of N-Carbon Quantum Dots " , *J. Lumin.*, 2018. https://doi.org/10.1016/j.jlumin.2018.04.061

[13] Y. Guo and W. Zhao, “Spectrochimica Acta Part A : Molecular and Biomolecular Spectroscopy Hydrothermal synthesis of highly fl uorescent nitrogen-doped carbon quantum dots with good biocompatibility and the application for sensing ellagic acid,” *Spectrochim. Acta Part A Mol. Biomol. Spectrosc.*, vol. 240, p. 118580, 2020. https://doi.org/10.1016/j.saa.2020.118580

[14] Vikas Kumar Singh,a Virendra Singh,b Pradeep Kumar Yadav,a Subhash Chandra,a Daraksha Bano,a Vijay Kumara, Biplob Koch,b Mahe Talat a and Syed Hadi HasanA. " Bright-blue-emission nitrogen and phosphorus doped carbon quantum dots as a promising nanoprobe for detection of Cr (VI) and ascorbic acid in pure aqueous solution and in living cell", New Journal of Chemistry 2018. DOI: 10.1039/C8NJ02126K

[15] G. Kalaiyarasan and J. Joseph, “Determination of vitamin B12 via pH-dependent quenching of the fluorescence of nitrogen doped carbon quantum dots,” pp. 3883–3891, 2017. DOI 10.1007/s00604-017-2421-y

[16] M. Wang, Y. Xia, J. Qiu, and X. Ren, “Carbon quantum dots embedded mesoporous silica for rapid fluorescent detection of acidic gas” *Spectrochim. Acta Part A Mol. Biomol. Spectrosc.*, 2018. doi:10.1016/j.saa.2018.08.006

[17] M. Wang, X. Ren, L. Zhu, Y. Xia, and J. Qiu, “Preparation of Microporous and Mesoporous Materials Preparation of mesoporous silica / carbon quantum dots composite and its application in selective and sensitive Hg 2 + detection,” *Microporous Mesoporous Mater.*, vol. 284, no. March 2018, pp. 378–384, 2019. https://doi.org/10.1016/j.micromeso.2019.04.026

[18] J. Lv, L. Fu, B. Zeng, M. Tang, and J. Li, “Synthesis and Acidizing Corrosion Inhibition Performance of N-Doped Carbon Quantum Dots,” vol. 92, no. 6, pp. 848–856, 2019. DOI: 10.1134/S1070427219060168

[19] N. Wang, Z. He at.al. “pH - sensitive carbon quantum dots − doxorubicin nanoparticles for tumor cellular targeted drug delivery,” vol. 12, no. May, pp. 1–10, 2019. DOI: 10.1002/pat.4696

[20] C. Zhao, X. Wang, L. Wu, W. Wu, Y. Zheng, and L. Lin, “Nitrogen-doped carbon quantum dots as an antimicrobial agent against Staphylococcus for the treatment of infected wounds,” *Colloids Surfaces B Biointerfaces*, vol. 179, no. February, pp. 17–27, 2019. https://doi.org/10.1016/j.colsurfb.2019.03.042

[21] M. Wang, Y. Jiao, C. Cheng, J. Hua, and Y. Yang, “Nitrogen-doped carbon quantum dots as a fluorescence probe combined with magnetic solid-phase extraction purification for analysis of folic acid in human serum,” pp. 7063–7075, 2017. DOI 10.1007/s00216-017-0665-3

[22] use: R. Tian, S. Zhong, J. Wu, Y. Gen, B. Zhou, Q. Wang and W. JiangA. " Facile preparation and the stepwise formation mechanistic investigation of gram-scale nitrogen-doped graphene quantum dots" J. Mater. Chem. C, 2017, DOI: 10.1039/C7TC02434G.

[23] H. J. Lee, J. Jana, Y. T. Ngo, L. L. Wang, J. S. Chung, and S. H. Hur, “The effect of solvent polarity on emission properties of carbon dots and their uses in colorimetric sensors for water and humidity,” *Mater. Res. Bull.*, vol. 119, no. May, p. 110564, 2019. https://doi.org/10.1016/j.materresbull.2019.110564

[24] S. Zhuo, L. Gao, P. Zhang, and J. Du, "Living cell imaging and sensing of hydrogen sulfide using high-efficiency fluorescent Cu-doped carbon quantum dots" pp. 19659–19664, 2018. DOI: 10.1039/c8nj03654c

[25] D. B. B. Siddlingeshwar, R. H. Krishna, V. Singh, N. Kottam, and D. Devang, "Green and Cost Effective Synthesis of Fluorescent Carbon Quantum Dots for Dopamine Detection," NewJ.Chem., 2018, 42, 19659 https://doi.org/10.1007/s10895-018-2218-3

[26] P. Supchocksoonthorn, A. K. Roy, W. Chae, et.al. "Microwave-Assisted Synthesis of Multifunctional Fluorescent Carbon Quantum Dots from A 4 / B 2 Polyamidation Monomer Sets," *Appl. Surf. Sci.*, p. 148471, 2020. https://doi.org/10.1016/j.apsusc.2020.148471

[27] D. Bharathi, R. H. Krishna, B. Siddlingeshwar, D. Devang and et. al. "Understanding the interaction of carbon quantum dots with CuO and Cu 2 O by fluorescence quenching," *J. Hazard. Mater.*, vol. 369, no. January, pp. 17–24, 2019. https://doi.org/10.1016/j.jhazmat.2019.02.008

[28] S. Zhuo *et al.*, "Facile fabrication of fluorescent Fe-doped carbon quantum dots for dopamine sensing and bioimaging application" *Analyst*, vol. 144, pp. 656–662, 2019. DOI: 10.1039/c8an01741g

[29] Y. Liang, Y. F. Shen, C. L. Liu, and X. Y. Ren, "Effects of chemical bonds between nitrogen and its neighbor carbon atoms on fl uorescence properties of carbon quantum dots," *J. Lumin.*, vol. 197, no. November 2017, pp. 285–290, 2018. https://doi.org/10.1016/j.jlumin.2018.01.034

[30] Shi, Y., Pan, Y., Zhong, J., Yang, J., Zheng, J., Cheng, J., Song, R., Yi, C., Facile synthesis of gadolinium (III) chelates functionalized carbon quantum dots for fluorescence and magnetic resonance dualmodal bioimaging, Carbon (2015), doi: http://dx.doi.org/10.1016/j.carbon.2015.05.100

[31] Han, T., Yan, T., Li, Y., Cao, W., Pang, X., Huang, Q., Wei, Q., Eco-friendly synthesis of electrochemiluminescent nitrogen-doped carbon quantum dots from diethylene triamine pentacetate and their application for protein detection, Carbon (2015), doi: http://dx.doi.org/10.1016/j.carbon.2015.04.053

[32] J. Zhou, T. Han, H. Ma, T. Yan, X. Pang, Y. Li, Q. Wei, A Novel Electrochemiluminescent Immunosensor Based on the Quenching Effect of Aminated Graphene on Nitrogen-Doped Carbon Quantum Dots, Analytica Chimica Acta (2015), doi: 10.1016/j.aca.2015.07.018.

[33] A. Gao, Y. Kang and X. Yin,A. "Red fluorescence-magnetic resonance dual-modality imaging applications of gadolinium-containing carbon quantum dots with excitation-independent emission" New J. Chem., 2017, DOI: 10.1039/C7NJ00597K.

[34] A. N. Tarasenka, A. Stupak, N. Tarasenko, D. Mariotti, and S. Chakrabarti, "Structure and optical properties of carbon nanoparticles generated by laser treatment of graphite in liquid." ChemPhysChem 10.1002/cphc.201601182

[35] H. Soni and P. Padmaja, "Liquid crystalline multilayer graphene quantum dots with hackelite structures : Characterisation and application for sensing nitrophenols," *Sensors Actuators B. Chem.*, vol. 268, pp. 100–107, 2018. https://doi.org/10.1016/j.snb.2018.04.116

[36] L. Wang *et al.*, "A ratiometric fluorescence and colorimetric dual-mode assay for H 2 O 2 and xanthine based on Fe , N co-doped carbon dots," vol. 180, no. April, 2020. https://doi.org/10.1016/j.dyepig.2020.108486

[37] Y. Shi, C. Li, S. liu, Z. Liu, J. Zhu, Y. Jidong and X. hu V. A. Online, C. Li, Z. Liu, J. Zhu, and Y. Jidong, "Facile synthesis of 1 fluorescent carbon dots for determination of curcumin based on fluorescence resonance energy transfer" 2015. RSC Adv., 2015, DOI: 10.1039/C5RA13404H
